# Chronic Rhinosinusitis with Nasal Polyps: A “Module-First” Review of Murine Models and Chemical Interventions

**DOI:** 10.3390/molecules31050781

**Published:** 2026-02-26

**Authors:** Yunfei Gao, Gengluan Liu, Caiyan An, Hesen Huang, Huaixiang Zhou, Junjing Zhang, Yunping Fan, Ningning Li

**Affiliations:** 1 Department of Otolaryngology, The Seventh Affiliated Hospital of Sun Yat-sen University, Shenzhen 518107, China; gaoyf25@mail.sysu.edu.cn (Y.G.); 18805067806@163.com (H.H.); 2 Tomas Lindahl Nobel Laureate Laboratory, The Seventh Affiliated Hospital of Sun Yat-sen University, Shenzhen 518107, China; liugluan@mail2.sysu.edu.cn (G.L.); zhouhuaixiang@sysush.com (H.Z.); 3 Department of Respiratory and Critical Care Medicine, The Seventh Affiliated Hospital of Sun Yat-sen University, Shenzhen 518107, China; 4 Inner Mongolia Key Laboratory of Allergic Diseases, Foundational and Translational Medical Research Center, Hohhot First Hospital, Hohhot 010030, China; acy_1999@163.com; 5 Department of Hepato-Biliary Surgery, Department of Surgery, Hohhot First Hospital, Hohhot 010030, China

**Keywords:** CRSwNP, murine models, molecular endotypes, tissue remodeling, targeted therapy, ferroptosis, metabolic reprogramming

## Abstract

Chronic rhinosinusitis with nasal polyps (CRSwNP) comprises multiple molecular endotypes that only partly align with the clinical phenotype, which complicates target selection and interpretation of treatment effects. Human omics and biomarker studies define candidate pathways, but causal attribution of specific nodes to lesion formation and remodeling requires perturbable in vivo systems. Here, we present a “module-first” framework that links murine induction paradigms to epithelial–immune–stromal circuits and to a minimal, module-matched endpoint set for reproducible causal inference. We summarize commonly used CRSwNP-like protocols (allergen/protease ± SEB, aeroallergen + SEB, innate trigger-enriched paradigms, and modifier layers), emphasize operational pathology terminology (“polyp-like lesion” versus “true polyp”), and propose a uniform causal template for validated pathway modules (alarmins/IL-33–NF-κB, type 2/ILC2–eosinophil, IL-17A/neutrophil, Wnt/EMT remodeling, and JAK/STAT kinase convergence). Finally, we organize chemical and molecular interventions by leverage point and propose an ARRIVE-aligned Minimum Reporting Set to standardize model anchoring, target engagement, and cross-study comparability. This module-first roadmap is intended to accelerate mechanism-linked discovery and preclinical validation of tractable drug targets in CRSwNP. Importantly, this module-first roadmap is intended as a heuristic organizing principle rather than an exhaustive taxonomy, because pathway modules can overlap and shift dynamically across time and tissue compartments in vivo.

## 1. Introduction: From Clinical Phenotypes to Targetable Modules

### 1.1. The Necessity of Murine Models: Causal Dissection of Molecular Endotypes

Chronic rhinosinusitis (CRS) is now recognized as a heterogeneous inflammatory disorder rather than a single entity defined by symptom duration or the presence of nasal polyps. Across cohorts, transcriptomic and immunologic profiling has resolved multiple endotypes (type 2, non-type 2, mixed, and remodeling-forward), with distinct effector programs and therapeutic vulnerabilities [1,2,3]. The clinical category of CRSwNP captures a high-burden subset, but it does not, by itself, specify the dominant pathway module driving mucosal lesion formation, persistence, and recurrence [1,2,4,5,6].

Human studies are indispensable for defining endotype signatures and for linking biomarkers to outcomes, but they are intrinsically observational. Tissue sampling is limited in depth and timing, and many readouts are correlative with confounding by treatment exposure, comorbid asthma/AERD, and microbial context. Murine models therefore remain essential to test causality: they allow controlled exposures, timed perturbations (genetic or pharmacologic), and standardized lesion scoring with on-target pathway validation [2,4,5].

A brief translational caveat is warranted at the outset. Murine sinonasal anatomy differs from humans (e.g., smaller paranasal sinuses, distinct gland density, and constrained obstruction/retention physiology), which limits direct modeling of ostial obstruction and symptom-defining mucus retention. Accordingly, structural and imaging readouts should be interpreted as standardized within-model quantification rather than direct surrogates of human disease severity; we discuss these constraints and reporting recommendations in Section 5.

### 1.2. The Epithelial–Immune–Stromal Triad: A Map of Druggable Circuits

We propose a “module-first” framework that treats CRSwNP as a dysregulated epithelial–immune–stromal circuit. In this view, controlled sinonasal exposures act on a compromised barrier to release epithelial “alarmins” (TSLP, IL-25, IL-33), which bias downstream immune modules (type 1, type 2, or type 3) and converge on a remodeling effector layer that sustains polypoid inflammation [7].

Epithelial interface: Barrier injury and impaired mucociliary clearance are proximal triggers that amplify alarmin signaling and facilitate persistent antigen–microbe interactions. Key readouts include tight-junction markers, mucociliary genes, and epithelial stress programs that precede immune polarization [1,2].

Immune modules: CRS spans type 1, type 2, and type 3 signatures, but eosinophilic CRSwNP is most often dominated by the type 2 module (IL-4/IL-5/IL-13 with Th2/ILC2 programs and eosinophilia). Biologics that target IL-4Rα (e.g., dupilumab; and next-generation IL-4Rα antibodies such as stapokibart in related upper-airway type 2 disease) illustrate how pathway-defined modules can guide intervention selection and mechanistic readouts [8,9].

Stromal remodeling: Remodeling is not a passive consequence. Altered fibrinolysis (e.g., tPA downregulation), excess fibrin deposition, and MMP/TIMP imbalance shape edema, extracellular-matrix turnover, and polypoid architecture, forming persistence loops that can be causally tested in vivo [3,10].

### 1.3. Strategic Roadmap

Model choice should be driven by the dominant module under study, the timing of perturbation (initiation versus maintenance), and a pre-specified minimal comparability set of endpoints. Throughout this review, we map induction paradigms to pathway modules and recommend module-matched on-target markers together with standardized lesion terminology and scoring rubrics [11]. We emphasize that the module-first framework is a heuristic map to improve selection and interpretability, not a claim that in vivo CRSwNP biology consists of discrete, non-overlapping pathways.

**Figure 1:**
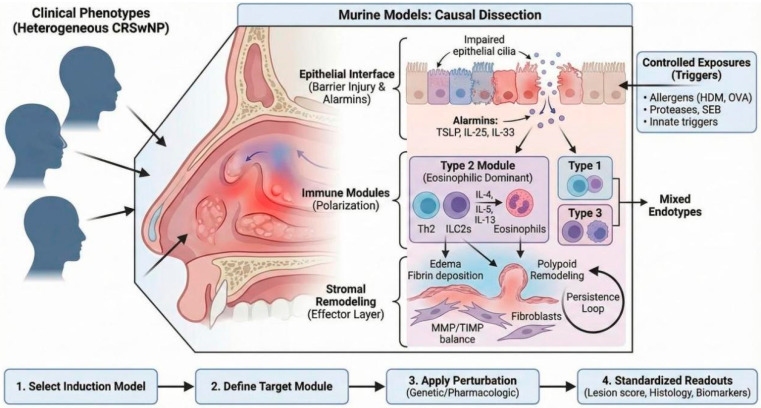
A module-first framework linking murine CRS/CRSwNP-like induction paradigms to targetable epithelial–immune–stromal circuits and standardized readouts. To read this figure, start from the left (clinical/endotype context) and follow the arrows to induction triggers and the dominant pathway module engaged; the bottom workflow indicates the intended sequence: select model → define module → apply perturbation → quantify with standardized readouts. Module-first framework for causal dissection of murine CRS/CRSwNP-like disease. Controlled sinonasal exposures (e.g., HDM/OVA, SEB/proteases, innate triggers) induce epithelial barrier injury and impaired mucociliary clearance, leading to alarmin release (TSLP, IL-25, IL-33) and engagement of immune polarization modules (type 2-dominant; type 1/type 3; mixed endotypes). These modules converge on a stromal remodeling effector layer characterized by edema, fibrin deposition/fibrinolysis imbalance, MMP/TIMP dysregulation, fibroblast activation, and polypoid remodeling with persistence loops. Bottom workflow: model selection → target module definition → timed perturbation (genetic/pharmacologic) → standardized readouts (lesion rubric, histology, biomarkers). Abbreviations: CRS, chronic rhinosinusitis; CRSwNP, chronic rhinosinusitis with nasal polyps; HDM, house dust mite; OVA, ovalbumin; SEB, staphylococcal enterotoxin B; TSLP, thymic stromal lymphopoietin; MMP, matrix metalloproteinase; TIMP, tissue inhibitor of metalloproteinases.

This review provides a rational framework for selecting murine models based on the specific molecular machinery they interrogate. Rather than categorizing models by induction method alone, we map them to the pathway modules they most robustly engage—from alarmin initiation to type 2/3 polarization and tissue remodeling [1,2,12]. We specifically highlight models mimicking recalcitrant eosinophilic disease and integrate endpoints relevant to chemical biology. Our approach follows a rigorous logic: select induction model → define target module → apply perturbation → quantify via module-matched readouts, thereby standardizing preclinical validation for next-generation therapeutics (Figure 1).

### 1.4. How to Use This Review (Practical Workflow)

This review is designed for readers who want to (i) select a murine induction paradigm that best engages a pathway module of interest, (ii) apply a genetic or chemical perturbation with interpretable target engagement, and (iii) report a minimal, comparable endpoint set. Practically, we suggest a three-step workflow: (1) define the question and the dominant module (alarmin initiation, type 2, type 3/neutrophilic, remodeling-forward, or kinase convergence); (2) choose an induction paradigm that most robustly engages that module (Table 1), avoiding extrapolation from type 2-dominant models when the question is non-type 2 biology; and (3) pre-specify readouts using the minimal comparability set (MCS) plus module-matched markers (lesion terminology and blinded scoring, granulocyte quantification, ≥1 remodeling biomarker, and ≥1 on-target engagement marker).

## 2. Murine CRSwNP-like Models as Causal-Testing Platforms

Murine models are essential for causal inference in CRSwNP because they permit controlled manipulation of (i) exposures (e.g., allergen, protease activity, superantigens, innate triggers), (ii) host susceptibility (genetic background or engineered alleles), and (iii) mechanism-linked interventions, enabling an evidence chain that cannot be assembled from human mucosal sampling alone [25]. Historically, CRS modeling in mice progressed from acute bacterial rhinosinusitis (e.g., intranasal *S. pneumoniae*) [13], to chronic bacterial inflammation [14], and subsequently to chronic eosinophilic sinonasal inflammation induced by repeated allergen or fungal extract challenges [15]. Building on these foundations, CRSwNP-oriented paradigms introduced epithelial “danger” features—most commonly protease activity and/or staphylococcal enterotoxin B (SEB)—to strengthen type 2 polarization and to elicit polypoid remodeling that is suitable for pathway-targeted testing [16,17,18,19,20].

### 2.1. Operational Pathology Definitions: “Polyp-like Lesion” vs. “True Polyp”

A major translational pitfall is that many murine “CRSwNP” protocols generate polypoid inflammation without consistently producing lesions that meet stringent criteria for a bona fide polyp. Recent validation work emphasizes that even widely used protocols may show substantial inflammatory remodeling yet fall short of true polyps, depending on lesion definitions and scoring thresholds [17].

#### 2.1.1. Polyp-like Lesion (Recommended Wording for Most Mouse Protocols)

A localized mucosal bulge characterized by edema/remodeling and inflammatory infiltrates on histology; typically small, nonpedunculated, and often best appreciated microscopically [16,17,18].

#### 2.1.2. True Polyp (Use Cautiously in Mice)

A discrete, protruding lesion that meets minimal reproducible histopathologic criteria: (i) a mucosal protrusion present across ≥3 serial sections at standardized anterior–posterior levels; (ii) a polypoid architecture with a stromal/edematous core covered by respiratory epithelium; (iii) a contour distinguishable from diffuse mucosal thickening; and (iv) reproducibility across animals and confirmation by blinded observers using a pre-specified scoring rubric. Concordant gross/endoscopic impression is encouraged when feasible [17].

Importantly, pathological rigor and translational utility are not mutually exclusive. Polyp-like lesions can still be used to address clinically relevant questions about barrier dysfunction, edema, granulocyte recruitment, and type 2 amplification under defined exposures. However, claims that depend on bona fide polyp architecture or recurrence-like behavior (e.g., polyp structural endpoints, pedunculated morphology, or statements about ‘true polyp’ formation) should be reserved for lesions that meet the above true-polyp criteria.

### 2.2. Induction Paradigms and Best-Use Cases (Endotype-Aligned)

#### 2.2.1. Type 2/Eosinophilic CRSwNP-like Inflammation: Allergen/Protease ± SEB

For type 2 (eosinophilic) CRSwNP-like disease, the most widely adopted strategy is allergen and/or protease exposure with optional SEB, which robustly engages alarmin–type 2 circuits and supports therapeutic testing.

OVA + SEB induces strong type 2 inflammation and polypoid remodeling and is frequently used for intervention studies (including topical pathway inhibitors). Unless strict “true polyp” criteria are met, outcomes should be described as polyp-like lesions [16,17].

HDM + SEB increases clinical realism by combining a common environmental allergen with superantigen exposure; it reproducibly induces type 2-skewed polypoid lesions in C57BL/6 contexts [18].

OVA + Aspergillus protease (AP) refines epithelial triggering because protease activity directly models barrier injury and alarmin release; this paradigm produces eosinophilic inflammation with polyp-like lesions within defined time windows [19].

Multiple airborne protease-active allergens provide trigger diversity and can be useful for robustness testing across exposure classes [20].

#### 2.2.2. Non-Type 2 (Type 3/Mixed) CRSwNP-like Inflammation: Innate Trigger-Enriched and EMT-Focused Paradigms

When the scientific question concerns neutrophilic, IL-17-associated, IFN-γ-associated, or mixed endotypes, it is preferable to use paradigms designed to engage these pathways rather than extrapolating from type 2-dominant models. In practice, this includes innate trigger-enriched protocols (e.g., LPS or Poly(I:C) exposures) discussed across CRS model surveys, and EMT-centered mechanistic designs that explicitly interrogate epithelial transition programs relevant to non-type 2 inflammation [25]. For example, an IFN-γ-linked kinase axis has been implicated in exacerbating neutrophilic CRS by promoting EMT, providing a rationale for EMT-aligned endpoints and perturbations in non-type 2 modeling [21]. Consistent with endotype frameworks, model choice and readouts should be matched to the dominant immune module being tested (type 2 vs. type 3/mixed) [2,12].

We further caution that type 2-derived readouts (e.g., eosinophil counts, IL-13-driven mucus signatures) should not be used as primary surrogates for non-type 2 biology. For non-type 2/neutrophilic contexts, recommended endpoints include neutrophil density (e.g., Ly6G+ cells), IL-17A/IFN-gamma axis markers, CXCL1/2, and epithelial injury/EMT panels. Chemical biology strategies that are particularly informative in these settings include pathway-selective modulation of TLR/TRIF signaling, CXCR2 or IL-17A-axis perturbation, and on-target engagement biomarkers matched to the hypothesized module.

#### 2.2.3. Modifiers and Comorbidity Modules: Severity, Refractoriness, and Remodeling Bias

Host-modifier layers can be integrated to probe why a model becomes severe or treatment-refractory. Vitamin D3 deficiency, for example, can exacerbate sinonasal inflammation and alter local vitamin D metabolism, supporting the concept that host nutritional/endocrine state modifies mucosal immune tone and remodeling propensity [23]. Remodeling bias modules can also be interrogated through pathways linked to epithelial EMT and tissue restructuring; Wnt signaling has been associated with EMT programs in CRSwNP, reinforcing EMT as a mechanistic bridge between inflammation and remodeling-oriented phenotypes [22]. In eosinophilic settings, periostin has been studied as a modulatory factor in murine CRSwNP-like disease, illustrating how stromal/ECM-associated mediators can shape inflammatory outcomes [26]. More broadly, systematic CRS model syntheses provide a framework for layering comorbid features or environmental modifiers to test generalizability of mechanism-linked interventions across contexts [25].

Translational pharmacology principle. Mouse intervention studies are most persuasive when (i) the model endotype matches the drug mechanism, and (ii) endpoints are pre-specified and objective (lesion definition, eosinophil/neutrophil quantification, remodeling markers, and blinded histologic scoring). A representative example is topical JAK inhibition evaluated in an eosinophilic CRSwNP model using polyp-like lesion counts and type 2 readouts as primary endpoints [24]. This mechanism-matched logic aligns with the biologics-era research agenda in CRSwNP: endotype definition → targeted intervention → objective endpoints [8,27].

Collectively, type 2-skewed CRSwNP-like paradigms (OVA + SEB, HDM + SEB, OVA + Aspergillus protease, and multi-allergen/protease exposures) and non-type 2/mixed or remodeling-forward designs (e.g., innate trigger-enriched and EMT-centered protocols, including IFN-γ-linked EMT models and Wnt/EMT-biased layers) provide endotype-aligned exposure contexts for mechanism-linked intervention testing. However, induction alone rarely resolves the cell-of-origin of a signal (e.g., epithelial alarmins) or identifies which compartment is necessary for sustaining a given module. Therefore, genetically engineered approaches layered onto these defined induction settings are required to convert associative phenotypes into compartment-resolved causal statements.

### 2.3. Transgenic Toolbox: From Association to Causality in Murine CRS/CRSwNP Models

Genetically engineered approaches enable compartment-resolved causal testing in CRS/CRSwNP-like models by combining (i) cell-type targeting (Cre drivers), (ii) lineage reporting (Cre-dependent reporters), and (iii) functional perturbation (conditional loss/gain of function or cell ablation). When integrated with defined induction paradigms (Section 2.2), these tools support attribution of alarmin release, immune polarization, granulocyte recruitment, mucus metaplasia, and remodeling to specific compartments.

Cre drivers and temporal control. Constitutive Cre lines can confound adult-onset inflammation by altering baseline tissue architecture, whereas tamoxifen-inducible CreER systems provide temporal control that is better suited for disease induction studies [28]. Because recombination efficiency varies by tissue and dosing, sinonasal studies should report tamoxifen regimen, washout period, and quantitative recombination estimates in the relevant compartment (e.g., flow cytometry, histology, or targeted PCR from sorted cells).

Lineage reporting and mapping. Cre-dependent reporters are essential for validating targeting specificity and recombination efficiency. The Rosa26 locus is a standard reporter landing site [29], and modern fluorescent reporters (e.g., tdTomato-based systems) enable sensitive detection of recombined cells and facilitate mapping of epithelial, immune, and stromal compartments [30]. Given that reporter sensitivity can alter the apparent specificity of a driver, studies should document gating/thresholding decisions and tissue processing workflows.

Functional perturbation: conditional genetics and ablation. Conditional alleles offer direct tests of pathway necessity in specified compartments. For “cell necessity” questions, Cre-inducible diphtheria toxin receptor (iDTR) enables lineage ablation after diphtheria toxin administration [31], while Cre-driven diphtheria toxin A (DTA) strategies provide robust ablation without systemic toxin delivery [32]. In sinonasal inflammation models, ablation studies should employ acute endpoints and stringent controls (toxin-only, Cre-only, and reporter-matched cohorts), because barrier disruption or systemic inflammatory effects can mimic target-cell phenotypes.

Common pitfalls and minimum control set. Conditional targeting studies are vulnerable to incomplete recombination, ectopic or variegated Cre activity, Cre toxicity, and background/microbiome effects; practical solutions and reporting expectations have been summarized and apply directly to sinonasal disease modeling [33]. In immune-driver settings, Cre activity may extend beyond the intended lineage depending on the driver and reporter sensitivity [34,35]. Therefore, for each new Cre driver in sinonasal contexts, studies should include (i) reporter-based mapping in nasal/sinonasal tissues, (ii) quantification of recombination in off-target leukocyte subsets, and (iii) explicit reporting of Cre allele, reporter allele, and genetic background.

Genetic tools strengthen causal inference, but they also introduce additional sources of variability, including recombination efficiency, off-target activity, background effects, and (for ablation strategies) systemic toxicity. Consequently, meaningful comparisons across laboratories require more than listing the induction protocol and allele. In the next section, we formalize a minimal comparability set (MCS) centered on a pre-specified lesion definition and scoring rubric, blinded histologic scoring, quantitative granulocyte endpoints, and at least one core remodeling biomarker aligned to the model.

### 2.4. Model Selection and Minimal Comparability Set (MCS)

Table 1 summarizes endotype-aligned induction paradigms and their best-use claims, whereas Table 2 outlines how genetic strategies can be paired with these paradigms to enable compartment-resolved causal tests. To ensure cross-study comparability, we recommend a minimal comparability set (MCS) that should be reported for any murine CRS/CRSwNP-like experiment: (i) a pre-specified lesion definition and scoring rubric (polyp-like vs. true polyp), (ii) blinded histologic scoring, (iii) quantitative granulocyte endpoints (eosinophils and/or neutrophils), and (iv) at least one core remodeling biomarker matched to the model (e.g., tPA/fibrin deposition and/or MMP/TIMP balance).

## 3. Pathway Modules Validated in Mice

**Figure 2:**
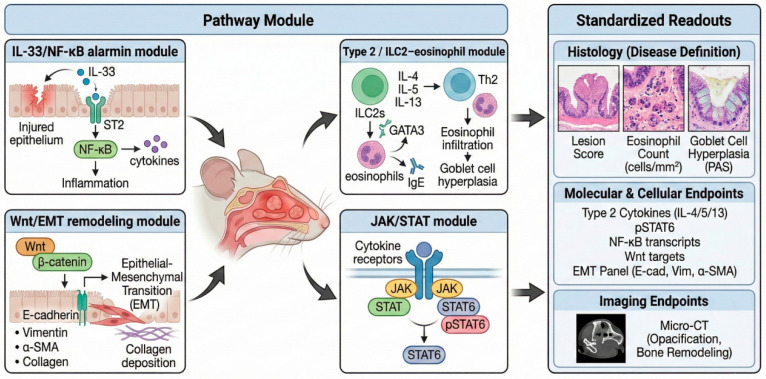
Endotype-aligned workflow linking murine induction paradigms to pathway modules and standardized readouts for causal inference in CRS/CRSwNP-like studies. To read this figure, follow the workflow from induction paradigm to dominant module, then to perturbation and the Minimal Comparability Set (MCS) plus module-matched panels for target engagement and phenotype quantification. Endotype-aligned pathway modules and standardized readouts in murine CRS/CRSwNP-like studies. To read this figure, follow the workflow from induction paradigm to dominant module, then to perturbation and the Minimal Comparability Set (MCS) plus module-matched panels for target engagement and phenotype quantification. Illustrated pathway modules include an IL-33/ST2–NF-κB alarmin module, a Type 2 (ILC2/Th2–eosinophil) module (IL-4/IL-5/IL-13; GATA3), a JAK/STAT kinase axis module (cytokine receptor signaling; pSTAT6), and a Wnt/EMT remodeling module (β-catenin; EMT markers; collagen deposition). Standardized readouts comprise histologic disease definition (lesion score; eosinophil counts; PAS goblet cell hyperplasia), module-matched molecular/cellular endpoints (e.g., Type 2 cytokines, NF-κB targets, EMT panel), and optional imaging endpoints (micro-CT for opacification/bone remodeling). Abbreviations: ILC2, group 2 innate lymphoid cell; NF-κB, nuclear factor kappa B; ST2, IL-33 receptor (IL1RL1); JAK, Janus kinase; STAT, signal transducer and activator of transcription; pSTAT6, phosphorylated STAT6; EMT, epithelial-to-mesenchymal transition; PAS, periodic acid–Schiff; micro-CT, micro-computed tomography.

We define ‘validated pathway modules’ in this review using three pragmatic criteria: (i) evidence of causal perturbation in murine CRS/CRSwNP-like settings (genetic or pharmacologic), (ii) availability of on-target engagement readouts that can be paired with a minimal disease endpoint set, and (iii) cross-species anchoring to human endotype biology. Several important processes (e.g., complement activation and neuro-immune signaling) are therefore discussed as cross-cutting amplifiers or candidate modules when the current murine evidence base is less uniform as a standalone module with a shared minimal endpoint set. The operational definition and evaluation workflow underlying this classification strategy are summarized in Box 1. The endotype-aligned validation workflow linking murine induction paradigms to pathway modules and standardized readouts is illustrated in Figure 2.

Box 1 **Uniform causal template for pathway modules (recommended).**

**Uniform causal template (5-field format).**

(1) Trigger/Model context: Specify the induction paradigm(s) and the endotype module engaged (type 2, non-type 2/mixed, remodeling-forward).
(2) Perturbation: State the causal manipulation (neutralization, inhibitor, conditional genetics/ablation) and timing window (initiation vs. maintenance).
(3) Mechanistic readouts (on-target): List 2–4 pathway-aligned markers demonstrating target engagement (e.g., axis markers, pSTAT, NF-κB targets).
(4) Phenotypic readouts (disease): Define lesion term and scoring rubric (polyp-like vs. true polyp), plus quantitative cellular/tissue endpoints (granulocytes, mucus, remodeling).
(5) Best-use claim: One sentence stating what this module is most credible for and what it should not be over-claimed for.

**Example (IL-33/NF-κB module):**

Trigger/Model context: OVA + SEB or HDM + SEB CRSwNP-like induction with alarmin-high epithelium.

Perturbation: Anti-IL-33 neutralization during induction/early maintenance [36].

Mechanistic readouts: IL-33/ST2 axis markers; NF-κB-aligned transcripts/cytokines [37].

Phenotypic readouts: Polyp-like lesion score, mucosal edema/thickness, inflammatory-cell density (blinded scoring; Table 1 notes).

Best-use claim: Causal test of “alarmin-to-effector” suppression with clear on-target readouts.

### 3.1. IL-33/NF-κB Alarmin Module

Trigger/Model. In allergen- and/or SEB-driven CRSwNP-like paradigms, epithelial alarmin release—particularly IL-33—functions as an upstream amplifier of mucosal inflammation.

Perturbation. Neutralizing IL-33 reduces inflammatory burden and attenuates polyp-like remodeling in murine CRS/CRSwNP-like settings, supporting IL-33 as an actionable upstream node rather than a passive biomarker [36].

Mechanistic readouts. Suppression of NF-κB-aligned inflammatory programs and downstream cytokine/chemokine expression; human CRSwNP studies further support an IL-33–NF-κB linkage [37].

Phenotypic readouts. Reduced polyp-like lesion score, mucosal thickness/edema, and inflammatory-cell density under blinded histologic scoring with pre-specified lesion definitions (see Table 1 notes).

Best-use claim. Best suited for testing “alarmin-to-effector” suppression strategies (e.g., anti-alarmin antibodies, NF-κB–pathway modulators, or epithelial-stabilizing interventions) with clear on-target readouts.

### 3.2. Type 2/ILC2–Eosinophil Module

Trigger/Model. Type 2-dominant CRSwNP-like models (e.g., HDM + SEB; OVA + AP) reproducibly induce IL-4/IL-5/IL-13 signatures with eosinophilic infiltration [18,19].

Perturbation. Prolonged allergen exposure sustains Th2-skewing and increases epithelial “alarmin tone” (e.g., TSLP), consistent with a maintenance-loop mechanism that is amenable to therapeutic disruption [38].

Mechanistic readouts. ILC2 activation and type 2 cytokine programs; human nasal polyp data showing ILC2 enrichment/activation provide a cross-species anchor for this module [39].

Phenotypic readouts. Quantitative eosinophil counts (cells/mm^2^ preferred; otherwise standardized HPF rules), goblet cell hyperplasia (PAS), and—when included—olfactory dysfunction endpoints as functional correlates [40,41].

Best-use claim. Primary platform for mechanism-matched testing of type 2-targeted interventions (biologics or small molecules), and for standardized comparisons of “type 2-dominant” versus “mixed” endotypes under consistent lesion nomenclature.

### 3.3. IL-17A/Neutrophilic Module

Trigger/Model. Non-type 2 CRS/CRSwNP-like inflammation can be modeled using IL-17A-biased paradigms and/or IFN-γ-linked inflammatory remodeling contexts that enrich neutrophilic features [21,42].

Perturbation. IL-17A blockade has been reported to mitigate disease features in experimental CRSwNP settings, supporting IL-17A as a causal effector node in neutrophilic/non-type 2 inflammation [42].

Mechanistic readouts. IL-17A-associated transcriptional programs and neutrophil trafficking markers; in selected models, IFN-γ–p38/ERK signaling aggravates remodeling via EMT-like epithelial changes [21].

Phenotypic readouts. Neutrophil quantification (Ly6G IHC preferred), epithelial injury indices, and lesion scoring explicitly labeled as “polyp-like” unless stringent “true polyp” criteria are met.

Best-use claim. Best suited to justify and test anti-type 3/Th17 strategies and to mechanistically explain why anti-type 2 monotherapy may underperform in mixed endotypes.

### 3.4. Wnt/EMT Remodeling Module

Trigger/Model. Wnt/β-catenin signaling is linked to EMT programs and remodeling-forward phenotypes relevant to CRSwNP pathobiology and experimental modeling [22].

Perturbation. In experimental settings, blockade of Wnt/β-catenin signaling attenuates EMT-associated signatures, indicating that remodeling circuitry can act as an active driver of persistence rather than merely a downstream consequence [22].

Mechanistic readouts. β-catenin/Wnt target genes together with an EMT marker panel (E-cadherin, vimentin, α-SMA) and complementary remodeling markers [22].

Phenotypic readouts. Collagen deposition (Masson/Sirius red), mucosal thickness, and lesion score/area fraction.

Best-use claim. Particularly suitable for positioning “anti-remodeling” small molecules as disease-modifying candidates—an angle that aligns well with *Molecules* readership.

### 3.5. JAK/STAT Module

Trigger/Model. Cytokine convergence on JAK/STAT provides a tractable kinase axis in murine CRSwNP-like inflammation.

Perturbation. Topical intranasal tofacitinib reduces polyp-like lesion burden and eosinophilic inflammation in mice, providing a clear proof-of-concept for local small molecule intervention [24].

Mechanistic readouts. The pSTAT signaling (context-dependent; commonly pSTAT6 in type 2 settings), tissue cytokines, and lavage biomarkers aligned to the targeted endotype [24].

Phenotypic readouts. Lesion score/count, eosinophil quantification, PAS mucus metrics, and remodeling markers as secondary endpoints.

Best-use claim. Strong “small molecule works in vivo” anchor that supports medicinal chemistry-friendly framing and mechanism-linked endpoint selection.

### 3.6. Readouts & Quantification Toolbox (Core + Module-Matched Panels)

#### 3.6.1. Histology as the Primary Disease-Definition Layer

Adopt the Table 1 rubric: pre-specified lesion terminology (polyp-like lesion vs. true polyp), serial-section confirmation when feasible, blinded scoring, and standardized cell-counting rules (cells/mm^2^ preferred; otherwise define HPF area and magnification).

#### 3.6.2. Imaging Endpoints to Reduce Observer Bias

Micro-CT provides a practical objective endpoint in murine models for longitudinal assessment of opacification and bone remodeling (including neo-osteogenesis) [43]. MRI/DWI can be mentioned as an optional translational adjunct for tissue characterization (e.g., edema–fibrosis separation and ADC-based metrics) rather than a routine murine standard [44].

#### 3.6.3. Molecular and Cellular Endpoints (“Module-Matched Panels”)

- Type 2 module: IL-4/IL-5/IL-13, GATA3, EPX/MBP; optional ILC2 flow/IHC where feasible [18,19,39].
- IL-33/NF-κB module: IL-33/ST2-axis markers and NF-κB-aligned transcripts [36,37].
- IL-17A/neutrophilic module: IL-17A, Ly6G/MPO, and IFN-γ-linked kinase readouts when relevant [21,42].
- Wnt/EMT module: β-catenin/Wnt targets plus EMT marker panel [22].
- JAK/STAT module: pSTAT readouts plus an endotype-matched cytokine panel [24].

#### 3.6.4. “Mechanistic Add-Ons” That Strengthen Causality Claims

Mitochondrial stress endpoints (e.g., epithelial mitochondrial morphology/function and mtROS) can be integrated when staphylococcal products/SEB are central to the hypothesis [45]. Human serum metabolomic signatures may support endotype alignment between cohorts and models but are best treated as supportive translational biomarkers rather than core murine endpoints [46].

## 4. Chemical and Molecular Interventions as Pathway Levers

Murine CRSwNP-like models have maximal translational value when used as causal-testing platforms, in which a defined perturbation (small molecule, biologic surrogate, or chemical probe) is applied to a defined endotype-aligned model, and efficacy is judged by target engagement together with core disease readouts (granulocytes, epithelial remodeling/mucus, stromal remodeling, and polyp-like lesion metrics). Consistent with the scope of *Molecules*, we organize interventions by molecular leverage points rather than conventional therapy classes. Throughout this section, chemical and natural product interventions are organized primarily as mechanistic probes/pathway levers to test necessity and target engagement in defined model contexts, rather than being presented as candidate therapies unless supported by human clinical evidence.

### 4.1. Small Molecules and Repurposed Drugs

Topical delivery is particularly informative in murine CRS/CRSwNP-like models because it enables sinonasal dose–response mapping while minimizing systemic confounding.

#### 4.1.1. Calcineurin/NFAT Inhibition (Cyclosporine, Intranasal)

Intranasal cyclosporine has been evaluated in eosinophilic polyp-like murine settings and was reported to reduce type 2 inflammation and polyp-like histopathology, supporting calcineurin/NFAT signaling as a tractable immunomodulatory axis for local intervention testing [47].

#### 4.1.2. JAK/STAT Axis (Tofacitinib, Intranasal)

Intranasal tofacitinib provides a local chemical perturbation to test the functional contribution of JAK/STAT signaling in type 2-skewed, polyp-like inflammation. Target engagement is best supported by pathway-aligned biomarkers (pSTAT signals; commonly pSTAT6 in type 2 contexts), alongside standardized lesion scoring and granulocyte quantification [24].

#### 4.1.3. Complement–Epithelial/Immune Amplification (C3aR Antagonism)

Complement signaling can act as an upstream amplifier in airway inflammation. In CRS-focused experimental work, pharmacologic C3a receptor antagonism has been proposed as an “upstream” lever to test whether complement activation is necessary for downstream cytokine programs and remodeling-associated phenotypes [48].

#### 4.1.4. Natural Products as Multi-Target Probes

Natural products can be positioned as polypharmacologic probes that perturb stress response and inflammatory networks. In a murine eosinophilic CRSwNP-like model, resveratrol was reported to attenuate sinonasal inflammation and polyp-like remodeling [49] In smoke-associated polyp-like inflammation, oridonin alleviated disease features through an autophagy-linked mechanism, providing a mechanistically framed “metabolism–stress response” lever rather than an empirical herbal observation [50]. These examples are discussed to illustrate pathway leverage and target engagement in vivo; given pleiotropy and context-dependence, they should be interpreted as mechanistic probes rather than direct therapeutic endorsements.

#### 4.1.5. Immunometabolic Control (GLUT1 Axis)

Targeting epithelial metabolic reprogramming represents an emerging direction. GLUT1-linked programs have been implicated in squamous metaplasia in eosinophilic CRSwNP, highlighting epithelial glucose transport/metabolic state as an intervenable module with direct histologic consequences [51]. Here, GLUT1-axis perturbation is presented as a mechanistic strategy to interrogate epithelial immunometabolism and remodeling, not as a definitive therapeutic recommendation.

### 4.2. Biologics as Molecular Tools (Murine Surrogates/Genetic Equivalents)

In mice, many human biologics show limited cross-reactivity; therefore, the experimentally appropriate framing is to use murine surrogate antibodies, receptor-blocking strategies, or genetic equivalents to test pathway necessity and compartmental contribution. Clinically successful biologics can be used as a human validation layer to prioritize which modules merit deeper mechanistic dissection and chemical biology exploration in mice.

Type 2 cytokine axis (IL-4/IL-13; IL-4Rα reference perturbation). Clinical efficacy of IL-4Rα blockade (dupilumab) supports the centrality of type 2 signaling in a substantial fraction of CRSwNP endotypes and provides a conceptual reference perturbation when mapping downstream chemistry (e.g., JAK/STAT convergence) and epithelial repair nodes [8].

IgE axis (anti-IgE as an endotype-dependent tool). Anti-IgE literature positions IgE blockade as an endotype-contextual probe to interrogate how local IgE class switching, mast cell activation, and epithelial danger signals integrate into polyp biology [52,53].

Upstream epithelial alarmins (TSLP blockade). TSLP inhibition (tezepelumab) represents a major upstream epithelial intervention axis. The WAYPOINT phase 3 study reported clinically meaningful improvements (including polyp size and congestion), and the U.S. FDA expanded the indication to include CRSwNP (age ≥ 12), making TSLP a high-priority pathway for back-translation into mouse causal modules using surrogates or genetic equivalents [54,55].

### 4.3. Chemical Probes and Imaging Readouts for Target Engagement

For a chemical biology framing, imaging and activatable probes function as quantitative readouts of target engagement and tissue consequences, rather than diagnostics.

(a) Micro-CT. Micro-CT enables objective quantification of sinonasal opacification and remodeling. In eosinophilic CRSwNP-like models, it has been used to evaluate osteitis/neo-osteogenesis and relate structural changes to inflammatory programs [43].
(b) MRI/DWI (optional translational adjunct). MRI-based approaches can support tissue characterization (e.g., differentiating edema-dominant from remodeling/fibrosis-oriented components) and may be positioned as a cross-species outcome measure in selected intervention studies, rather than a routine murine endpoint [44].
(c) Activatable near-infrared (NIR) probes for MMP activity. Because protease activity is a functional remodeling readout, MMP-activatable NIR probes provide a chemical-probe strategy for longitudinal monitoring of remodeling dynamics. Established activity-based probe frameworks support this approach, and CRSwNP remodeling literature implicates MMP dysregulation (including MMP-9) as relevant biology that can be interrogated by activity-sensitive imaging [11,56]. To facilitate experimental design and cross-study comparison, these pathway-directed interventions are summarized in Table 3.

## 5. Translational Alignment, Gaps, and Reporting Standards

### 5.1. Key Translational Gaps and Next-Generation Model Design Principles

(i) Chronicity is approximated rather than reproduced. Most CRSwNP-like mouse protocols achieve robust inflammation within weeks, but they rarely capture the relapsing course, long-lived immune imprinting, and therapy-shaped trajectories typical of human disease. Extending exposure duration improves face validity (e.g., prolonged allergen challenge), yet “polyp-like” pathology often persists and stringent “true polyp” criteria may still not be met without a pre-specified rubric and validation across observers [17,38].
(ii) Anatomical constraints limit obstruction- and sinus-centric biology. Murine sinonasal anatomy constrains modeling of sinus ostial obstruction, mucus retention, and gland-rich mucosa—features that are central to symptom burden and surgical endpoints in humans. Consequently, cross-species interpretation of structural readouts requires caution, and imaging endpoints should be framed as standardized quantification tools within the model rather than direct surrogates of human disease severity [25,43].
(iii) Exposure complexity is under-modeled. Human CRSwNP typically reflects layered and evolving exposures (allergens, microbial products, pollutants/irritants), whereas many murine studies still rely on one dominant trigger (±a single adjuvant such as SEB or protease activity). This limits external validity for mixed endotypes and for refractory disease questions; prioritizing multi-trigger or modifier-layer designs can improve robustness of mechanism-linked conclusions [25,27].
(iv) Key comorbidity modifiers are inconsistently integrated. Asthma and aspirin sensitivity/AERD-like features frequently co-occur with CRSwNP and can shape endotype expression and therapeutic response in patients. However, many murine platforms do not explicitly incorporate lower-airway inflammation or clinically relevant modifier layers when making translational claims. Where comorbidity modules are central to the hypothesis, they should be modeled explicitly and reported as such [2,4,5].
(v) Outcomes remain histology-heavy, with limited functional anchoring. Many mouse studies prioritize cytokines and cell counts but under-report functional correlates. Where feasible, adding validated olfactory behavioral assays strengthens translational alignment because olfactory dysfunction is clinically meaningful and has mechanistic links to mucosal inflammation in murine CRS/CRSwNP-like settings [40,41].

### 5.2. Reporting Standards for Molecules (ARRIVE 2.0)

For *Molecules*, reviewer concerns more often center on reproducibility and causal interpretability than on the nominal choice of model. Missing details on randomization/blinding, sectioning strategy, lesion definitions (polyp-like vs. true polyp), and quantitative denominators (cells/mm^2^ vs. cells/HPF) commonly trigger major revision. We therefore recommend explicitly stating compliance with ARRIVE 2.0 and providing a compact Minimum Reporting Set (MRS)—as a box in Methods or, at minimum, as a structured supplementary checklist (Table S1). This MRS should be aligned to the lesion/scoring logic used in Table 1 to support cross-study benchmarking [57,58].

### 5.3. Minimum Reporting Set (MRS)

To improve reproducibility and model comparability across laboratories, we propose an MRS spanning five domains: (A) animals and study design; (B) induction protocol; (C) tissue processing and lesion definition; (D) core readouts; and (E) statistics/transparency (Table S1).

A. Animals and study design (mandatory). Report strain/vendor, genetic background, engineered alleles/transgenes with genotype confirmation, sex, age (weeks), weight range, housing, and microbiological status (if known). Specify randomization method and blinding (who was blinded for dosing, scoring, quantification, and analysis). Provide sample size rationale, exclusion criteria, and attrition [57,58].

B. Induction protocol (mandatory). For each inducer (e.g., OVA + AP, HDM + SEB, IFN-γ/EMT-linked paradigms, smoke-associated protocols), report mass/concentration, volume per nostril, vehicle, route (instillation vs. aerosol vs. systemic sensitization), and frequency/duration. Include a timeline diagram (sensitization–challenge–endpoint) and clearly define the intervention window (prevention vs. treatment/reversal) [18,19,20,21,22,23,25].

C. Tissue processing and lesion definition (mandatory). Specify anatomical landmarks and anterior–posterior sectioning levels, number of levels per mouse, spacing between analyzed sections, and which levels were scored. Pre-specify operational definitions of “polyp-like lesion” vs. “true polyp” using an explicit rubric and positivity thresholds; report staining and quantification rules, including antibody identifiers and validation notes when IHC/IF is used [16,17,18].

D. Core readouts (recommended minimum set). Quantify eosinophils and/or neutrophils using a defined denominator (cells/mm^2^ preferred; if HPF is used, report HPF area and magnification), with field-selection rules and number of fields/levels per mouse. Include at least one endotype-matched cytokine panel (type 2 vs. IL-17A/neutrophilic) and ≥1 remodeling marker appropriate to the modeled module (e.g., EMT panel or collagen/ECM indices where relevant). If imaging is used, standardize acquisition and operational definitions (micro-CT for opacification/neo-osteogenesis; MRI/DWI as an optional adjunct when explicitly justified). Functional endpoints—especially olfactory assays—are strongly encouraged when feasible [39,40,41,42,43,44].

E. Statistics and transparency (mandatory). Declare the unit of analysis (mouse vs. section vs. field). Address nested data structures appropriately when multiple sections/fields per mouse are analyzed. Specify multiplicity correction when relevant. Ensure data availability for raw counts, scoring sheets, and representative full-section images.

A concise Methods statement (optional):

“All in vivo experiments and reporting followed ARRIVE 2.0 recommendations, including randomization, blinding of histologic scoring, and complete reporting of exclusions and sample size rationale.” [57,58].

## 6. Conclusions and Future Directions

Murine CRSwNP-like models have progressed from descriptive inflammation systems to mechanistically tractable platforms for causal testing of epithelial–immune–stromal circuits. The key limitation is no longer how to induce disease features, but how to standardize model anchoring, lesion definitions, quantitative endpoints, and reporting so that results can be compared across laboratories and aligned with human endotype biology. Moving forward, studies should adopt a module-first design—linking a defined induction context to an intervenable pathway module and validating causality with orthogonal perturbations and target engagement biomarkers—while applying strict polyp-like versus true-polyp terminology and blinded, quantitative scoring. Incorporating exposure complexity and clinically relevant modifier layers, when translational claims are made, will further strengthen external validity. Finally, routine use of an ARRIVE-aligned Minimum Reporting Set (MRS) should become the default to ensure reproducibility and enable cross-study benchmarking. If these principles are adopted, murine models can more reliably bridge pathway hypotheses to testable interventions, particularly in chemical biology frameworks that require precise target engagement and quantitative phenotyping [17,25,57,58].

Looking forward, modules with strong cross-species anchoring and tractable target engagement metrics (e.g., epithelial alarmin/NF-kappaB and type 2/ILC2-eosinophil circuits) appear most ready for near-term translational testing, whereas non-type 2 and mixed endotypes remain less mature and require improved model anchoring. The largest unmet methodological needs include standardized lesion rubrics (polyp-like vs. true polyp), serial-section reporting, blinded quantitative scoring, and routine inclusion of target engagement biomarkers to enable cross-study benchmarking and chemical biology reproducibility.

## Figures and Tables

**Table 1:** Endotype-aligned murine CRS/CRSwNP-like induction paradigms and best-use cases.

Category	Model (Mouse)	Induction (Core Components)	Duration	Dominant Endotype	Recommended Lesion Term *	Best Used For	Key Caveats
CRS-like (non-polypoid)	Acute bacterial rhinosinusitis	Intranasal *S. pneumoniae*	days	Type 1/innate	none	Acute host–pathogen responses	Not chronic CRS/NP biology [13]
	Chronic bacterial CRS	Obstruction ± bacterial persistence	weeks	mixed/innate	CRS-like	Chronic infection frameworks	Technical variability; not NP-focused [14]
	Chronic eosinophilic CRS (non-NP focus)	Repeated allergen/fungal challenge	~12 w	Type 2/eos	eosinophilic CRS-like	Chronic eosinophilic inflammation/remodeling	Often no polypoid lesions [15]
Type 2 CRSwNP-like	OVA + SEB	OVA sensitization/challenge + intranasal SEB	6–8 w	Type 2-skewed	polyp-like lesion	Type 2 loops; screen topical inhibitors	“True polyp” often unmet; strict rubric + blinding [16,17]
	HDM + SEB	HDM + intranasal SEB (often C57BL/6)	6–8 w	Type 2-skewed	polyp-like lesion	Higher exposure realism; mast cell/IgE layers	Strain/protocol sensitive; standardize dosing/scoring [18]
	OVA + Aspergillus protease (AP)	Protease-active epithelial injury + allergen	~10–12 w	Type 2/eos	polyp-like lesion	Barrier/alarmin-driven type 2 biology	Protease batch/activity QC required [19]
	Multiple protease-active allergens	Mixed airborne protease-active allergens	≥12 w	Type 2-biased	polyp-like lesion	Robustness across triggers	Exposure composition standardization is hard [20]
Non-type 2/remodeling-forward	IFN-γ/neutrophilic CRS-like	IFN-γ-linked kinase axis; EMT emphasis	varies	Type 1/3-like	neutrophilic CRS-like	Non-type 2 biology; EMT-linked refractoriness	Not a type 2 CRSwNP substitute [21]
	EMT/Wnt remodeling module	Wnt axis bias toward EMT/remodeling	varies	remodeling-forward	remodeling-forward CRS-like	Remodeling mechanisms; EMT endpoints	Avoid “polyp” labeling; focus on mechanistic endpoints [22]
Modifiers/use case	VD3 deficiency (modifier layer)	VD3-deficient diet + base model challenge	weeks	amplifies type 2	depends on base model	Host susceptibility; remodeling bias	Systemic confounding; diet control + matched base model [23]
	Use case: topical JAK inhibitor	Eosinophilic CRSwNP-like model + intranasal tofacitinib	weeks	Type 2	polyp-like lesion	Endpoint rigor + target engagement demonstration	Pre-specified endpoints; blinding/scoring critical [24]

Notes: Lesion terminology should follow Section 2.1; “polyp-like lesion” is recommended for most murine protocols unless stringent “true polyp” criteria and a pre-specified rubric are met [17]. * Lesion terminology should follow Section 2.1; in most murine protocols, “polyp-like lesion” is preferred unless stringent “true polyp” criteria and a pre-specified scoring rubric are met [22]. Abbreviations: AP, Aspergillus protease; CRS, chronic rhinosinusitis; CRSwNP, chronic rhinosinusitis with nasal polyps; EMT, epithelial-to-mesenchymal transition; eos, eosinophils; HDM, house dust mite; IFN-γ, interferon gamma; JAK, Janus kinase; neu, neutrophils; NP, nasal polyp; OVA, ovalbumin; SEB, staphylococcal enterotoxin B; VD3, vitamin D3; w, weeks.

**Table 2:** Genetics toolbox × model pairing to enable compartment-resolved causal tests in murine CRS/CRSwNP-like studies.

Genetic Strategy	Target Compartment	Best-Matched Models (Table 1)	On-Target Validation (Minimum)	Primary Readouts (Minimum)	Essential Controls/Pitfalls	Key Refs.
Inducible CreER/CreERT2 (timed recombination)	epi/immune/stroma	Any model where timing matters	Tamoxifen regimen + washout; quantified in sinonasal tissue	Lesion term + histology + granulocytes + on-target biomarker	Tamoxifen effects; recombination; report strain/sex/age	[28,33]
Rosa26 Cre reporter (baseline mapping)	epi/immune/stroma	Any Cre-based design	Reporter mapping in sinonasal tissue (IF/flow)	Compartment localization + recombination %	Reporter sensitivity affects “specificity”; document gating/thresholds	[29,30]
GOI^fl/fl^ × epithelial Cre(ER) (necessity test)	epithelium	Type 2 CRSwNP-like models	Reporter-confirmed epithelial recombination; GOI loss by qPCR/IF	Alarmins ± downstream type 2 readouts + lesion term	Do not infer from lung; include Cre–tamoxifen controls	[28,29,30,33]
GOI^fl/fl^ × lymphoid Cre(ER) (necessity test)	lymphoid	Type 2 CRSwNP-like models	Sorted-cell confirmation of GOI loss	Type 2 cytokines; eos counts; mucus/metaplasia	Developmental confounding if constitutive; off-target subset mapping	[28,29,30,33]
GOI^fl/fl^ × stromal/fibroblast-enriched CreER (persistence)	stroma	Type 2 CRSwNP-like; remodeling-forward	Reporter mapping in lamina propria; GOI loss in stromal fraction	tPA/fibrin axis; ECM markers; MMP/TIMP	Fibroblast heterogeneity; patchy recombination	[28,29,30,33]
iDTR + diphtheria toxin (cell necessity)	lineage-defined	Best for established-lesion “maintenance” tests	Local depletion efficiency quantified (IF/flow)	Pre/post lesion burden + module endpoints	DT systemic effects; DT-only controls (Cre+ iDTR + DT)	[31]
Rosa26-DTA (Cre-induced ablation)	lineage-defined	Mechanistic necessity (no systemic DT)	Depletion confirmed; injury markers monitored	Acute remodeling/lesion change + module endpoints	Irreversible; secondary injury inflammation; timing critical	[32]
Immune-driver specificity benchmarking	immune	Any immune Cre design	Reporter mapping across leukocyte subsets	Off-target rate estimate	Cre activity may extend beyond intended lineage	[34,35]
Orthogonal perturbations (genetic + pharmacologic)	any	Use case row; mechanism tests	Target engagement marker (e.g., pathway phosphorylation)	Lesion + cellular + biomarker triangulation	Avoid single-endpoint claims; pre-specify endpoints/blinding	[24,33]

Notes: GOI, gene of interest. For CreER systems, include tamoxifen vehicle and Cre− littermate controls. For ablation, include toxin-only controls and systemic toxicity monitoring. Examples of compartment drivers (report explicitly): epithelium (Krt5-, Foxj1-based), immune (LysM-, CD4-, CD11c-based), stroma/fibroblast (Col1a2-, Pdgfra-based). Abbreviations: CreER/CreERT2, tamoxifen-inducible Cre recombinase; DTA, diphtheria toxin A; DT, diphtheria toxin; iDTR, inducible diphtheria toxin receptor; IF, immunofluorescence; MMP, matrix metalloproteinase; TIMP, tissue inhibitor of metalloproteinases; tPA, tissue plasminogen activator.

**Table 3:** Chemical and molecular interventions as pathway levers in murine CRS/CRSwNP-like studies.

Intervention/Tool	Molecular Leverage Point	Best-Matched Models (Tag)	On-Target Engagement	Primary Disease Endpoints (MCS + Endpoints)	Key Caveats	Key Refs
Intranasal cyclosporine	Calcineurin/NFAT immunomodulation	Type 2	NFAT-pathway suppression (if available); reduced type 2 cytokine tone	Polyp-like lesion rubric + blinded histology; eos counts; mucus/remodeling markers	Local dosing critical; report vehicle controls and exposure protocol	[47]
Intranasal tofacitinib	JAK/STAT kinase convergence	Type 2	pSTAT (often pSTAT6 in type 2 contexts); endotype-matched cytokine panel	Polyp-like lesion score/count; eos counts; PAS mucus; ± remodeling	Confirm local target engagement; avoid single-endpoint claims	[24]
Complement-driven C3aR antagonism	Epithelial/immune amplification	Type 2; Non-type 2	C3aR pathway blockade markers (context-dependent)	Lesion rubric; granulocytes; remodeling marker matched to model	Effect size likely trigger-dependent; emphasize model–phenomenology	[48]
Resveratrol	Multi-target anti-inflammatory/anti-oxidative probe	Type 2	Network modulation markers (avoid single-target over-claim)	Lesion rubric; eos; mucus/remodeling readouts	Mechanism model-dependent; polypharmacology; interpret constrained	[49]
Oridonin	Stress response/autophagy-linked lever (smoke-associated)	Non-type 2	Autophagy-linked markers (as reported)	Squamous metaplasia indices + lesion rubric; epithelial markers; granulocytes	Trigger-specific (smoke); interpret within exposure	[50]
GLUT1-axis targeting (conceptual)	Epithelial immunometabolic reprogramming	Type 2; Remodeling-forward	GLUT1/metabolic markers (as reported)	Squamous metaplasia indices + lesion rubric; eos; epithelial markers	Emerging axis; standardize histology definitions	[51]
IL-4Rα axis (clinical validation layer)	Type 2 pathway neutralization (IL-4/IL-13)	Type 2	In mice: surrogate/genetic equivalents	Lesion rubric; eos; type 2 cytokines; mucus	Human biologics may not bind murine targets; frame as prioritization	[8]
Anti-IgE axis (clinical validation layer)	IgE/mast cell integration (endotype-dependent)	Type 2	In mice: surrogate equivalents; IgE/mast endpoints	Lesion rubric; eos + mast cell endpoints; mucus	Strong endotype dependence; avoid over-generalization	[52,53]
TSLP blockade (clinical validation layer)	Upstream epithelial alarmin axis	Type 2	In mice: surrogate/genetic equivalents; alarmins/type 2 panel	Lesion rubric; eos; alarmins/type 2 panel; ±remodeling marker	High priority for human translation; needs correct model match	[54,55]
Quantification tool: Micro-CT	Objective structural outcome (opacification; osteitis)	Remodeling-forward	Imaging-based quantitative scores	Opacification/bone remodeling indices; link to inflammation panels	Use as quantitative outcome, not “diagnostics”	[43]
Quantification tool: MRI/DWI (optional adjunct)	Tissue characterization (edema vs. remodeling components)	Remodeling-forward	ADC/DWI-derived indices (as proposed)	Distinguish inflammation vs. remodeling responses	Optional translational add-on; not routine	[44]
Quantification tool: Activatable NIR MMP probe (chemical probe)	Functional remodeling readout (MMP activity)	Remodeling-forward	Activity-based signal change (longitudinal)	Remodeling dynamics activity + lesion rubric + histology	Specify probe controls; interpret alongside MMP biology	[11,56]

Notes: For intervention studies, report the minimal comparability set (MCS): pre-specified lesion definition/rubric (polyp-like vs. true polyp), blinded histologic scoring, quantitative granulocyte endpoints, and ≥1 remodeling biomarker matched to the model (Section 2.4; Table 1 notes). Abbreviations: ADC, apparent diffusion coefficient; C3aR, complement C3a receptor; CRSwNP, chronic rhinosinusitis with nasal polyps; DWI, diffusion-weighted imaging; JAK, Janus kinase; MCS, minimal comparability set; MMP, matrix metalloproteinase; MRI, magnetic resonance imaging; NIR, near-infrared; NFAT, nuclear factor of activated T cells; PAS, periodic acid–Schiff; pSTAT, phosphorylated STAT; TSLP, thymic stromal lymphopoietin.

## Data Availability

No new data were created or analyzed in this study. Data sharing is not applicable to this article.

## Supplementary Materials

The following supporting information can be downloaded at: https://www.mdpi.com/article/10.3390/molecules31050781/s1, Table S1: Minimum Reporting Set (MRS) for murine CRS/CRSwNP-like studies. Table S2: Consolidated Abbreviations/Glossary.
