# Supplementary material for: Chronic Rhinosinusitis with Nasal Polyps: A “Module-First” Review of Murine Models and Chemical Interventions
Source: Molecules. 2026 Feb 26;31(5):781. doi: 10.3390/molecules31050781 (PMC12985795; doi:10.3390/molecules31050781)
Supplement: Supplementary file 1 [file molecules-31-00781-s001.zip › Table S1.pdf]

Table S1. Minimum Reporting Set (MRS) for murine CRS/CRSwNP-like studies aligned with Table 1 notes.

Domain	Item	Must report	Recommended	Minimum specification (what to write)	Align to Table 1 note
A. Animals & study design	Species/strain/vendor	√		Mouse strain, vendor, microbiological status if known	
	Genetic background/alleles	√		Background strain; Cre/reporter/toxin/DTR/DTA alleles; genotype confirmation method	
	Sex/age/weight	√		Sex; age (weeks); body-weight range at baseline	
	Housing/husbandry	√		SPF status if known; cage density; light/dark cycle; diet; enrichment	
	Group allocation	√		Randomization method (how assigned); allocation concealment if used	
	Blinding	√		Who was blinded for dosing, histology scoring, quantification, and analysis	TI-2
	Sample size rationale	√		Power calc or justified effect size; planned n/group	
	Exclusions/attrition	√		Pre-specified exclusion criteria; attrition and reasons	
	Unit of analysis declared	√		Mouse vs section vs field; how many sections/fields per mouse	TI-4
B. Induction protocol	Inducer(s) and rationale	√		Model name + trigger logic (e.g., OVA+SEB; HDM+SEB; OVA+AP)	TI-1
	Dose/concentration/vehicle	√		Mass + concentration; vehicle; batch info if relevant (e.g., protease activity)	
	Route and volume	√		IN instillation/aerosol/systemic sensitization; volume per nostril	
	Frequency/duration	√		Challenge schedule; total weeks; endpoints	
	Timing figure		√	Sensitization–challenge–endpoint schematic; intervention window marked	
	Comorbidity/modifier modules	√*	√	If used: asthma/airway/AERD-like; smoke; VD3 deficiency	
C. Tissue processing & lesion definition	Anatomical landmarks for sectioning	√		Defined anterior–posterior levels; nasal cavity map/landmarks	TI-3
	Sections per mouse & spacing	√		Levels scored; interval between analyzed sections; fields used for quantification	TI-3
	Lesion terminology	√		Explicitly state polyp-like lesion vs true polyp; pre-specified rubric	TI-1
	Positivity thresholds	√		Minimum section number criteria; how "positive lesion" defined	TI-1
	Histology stains	√		H&E (+ as applicable), PAS, Masson/Sirius red; protocol refs	TI-5
	IHC/IF antibody reporting	√		Antibody ID (vendor, catalog, clone), dilution, antigen	

Domain	Item	Must report	Recommended	Minimum specification (what to write)	Align to Table 1 note
				retrieval, controls	
D. Core readouts (MCS + module-matched)	Blinded scoring rubric	√		Who scored; scoring sheet; inter-observer if applicable	TI-2
	Granulocytes quantified	√		Eosinophils and/or neutrophils with denominator	TI-4
	Denominator & counting rules	√		Cells/mm² preferred; if HPF: report HPF area + magnification; fixed field selection	TI-4
	Endotype-matched cytokine panel		√	Type 2 (IL-4/IL-5/IL-13 ± TSLP) or non-type 2 (IL-17A/IFN-γ) aligned to model	
	Mucus/metaplasia		√	PAS goblet cell index or mucus gene markers; standardized quantification	TI-5
	Remodeling marker (≥1)	√		Choose ≥1: collagen/ECM (Masson/Sirius), EMT panel, or fibrin/tPA axis	TI-5
	Imaging endpoint (if used)	√*	√	Acquisition + reconstruction; operational definition of opacification/remodeling	TI-6
	Micro-CT reporting (if used)	√*	√	Scanner settings; segmentation; scoring definition; osteitis/co-remodeling metrics	TI-6
	MRI/DWI reporting (if used)	√*	√	Sequence; b-values; ADC derivation; tissue interpretation rules	TI-6
	Functional correlates		√	Olfactory behavioral assay (preferred) ± airflow proxies; protocol + scoring	
E. Statistics & transparency	Statistical plan	√		Tests used; effect size + CI where relevant; alpha; software	
	Nested data handling	√		How multiple sections/fields per mouse were modeled/aggregated	TI-4
	Multiple comparisons	√*		Correction approach when multiple endpoints/groups	
	Data availability	√		Raw counts, scoring sheets, representative full-section images per group	
	ARRIVE 2.0 statement		√	Explicit compliance statement in Methods/Supplement	[57,58]

* "Must report" applies when the item is used/claimed in the study.

TI legend (Table 1 notes): TI-1 lesion terminology/rubric; TI-2 blinding; TI-3 sectioning landmarks/spacing; TI-4 granulocyte quantification + unit of analysis; TI-5 remodeling markers/stains; TI-6 imaging endpoints.

Abbreviations: AERD, aspirin-exacerbated respiratory disease; DTA, diphtheria toxin A; DTR/iDTR, (inducible) diphtheria toxin receptor; HPF, high-power field; IF, immunofluorescence; IHC, immunohistochemistry; IN, intranasal; MCS, minimal comparability set; MRS, minimum reporting set; MRI, magnetic resonance imaging; OVA, ovalbumin; PAS, periodic acid–Schiff; SEB, staphylococcal enterotoxin B; tPA, tissue plasminogen activator; VD3, vitamin D3.
