# Supplementary material for: Chronic Rhinosinusitis with Nasal Polyps: A “Module-First” Review of Murine Models and Chemical Interventions
Source: Molecules. 2026 Feb 26;31(5):781. doi: 10.3390/molecules31050781 (PMC12985795; doi:10.3390/molecules31050781)
Supplement: Supplementary file 1 [file molecules-31-00781-s001.zip › Table S2.pdf]

**Supplementary Table S2. Consolidated Abbreviations/Glossary.**

Abbreviations used in the manuscript are listed alphabetically.

| Abbreviation | Definition |
|---|---|
| ADC | apparent diffusion coefficient |
| AERD | Aspirin-exacerbated respiratory disease |
| AP | Aspergillus protease |
| ARRIVE | Animal Research: Reporting of In Vivo Experiments |
| C3aR | complement C3a receptor |
| CreER/CreERT2 | tamoxifen-inducible Cre recombinase |
| CRS | chronic rhinosinusitis |
| CRSwNP | chronic rhinosinusitis with nasal polyps |
| DT | diphtheria toxin |
| DTA | diphtheria toxin A |
| DWI | diffusion-weighted imaging |
| EMT | epithelial-to-mesenchymal transition |
| eos | eosinophils |
| HDM | house dust mite |
| iDTR | inducible diphtheria toxin receptor |
| IF | immunofluorescence |
| IFN-γ | interferon gamma |
| IL1RL1 | IL-33 receptor |
| ILC2 | group 2 innate lymphoid cell |
| JAK | Janus kinase |
| MCS | minimal comparability set |
| micro-CT | micro-computed tomography |
| MMP | matrix metalloproteinase |
| MRI | magnetic resonance imaging |

| | |
|---|---|
| MRS | Minimum Reporting Set |
| neu | neutrophils |
| NF-κB | nuclear factor kappa B |
| NFAT | nuclear factor of activated T cells |
| NIR | near-infrared |
| NP | nasal polyp |
| OH | Dietary Vitamin D3 Deficiency Exacerbates Sinonasal Inflammation and Alters Local 25 |
| OVA | ovalbumin |
| PAS | periodic acid–Schiff |
| pSTAT | phosphorylated STAT |
| pSTAT6 | phosphorylated STAT6 |
| SEB | staphylococcal enterotoxin B |
| ST2 | IL-33 receptor (IL1RL1) |
| STAT | signal transducer and activator of transcription |
| SZSM202111005 | This work was supported in part by the Sanming Project of Medicine in Shenzhen |
| TIMP | tissue inhibitor of metalloproteinases |
| tPA | tissue plasminogen activator |
| TSLP | thymic stromal lymphopoietin |
| VD3 | vitamin D3 |
| w | weeks |
